# The expression of ASAP3 and NOTCH3 and the clinicopathological characteristics of adult glioma patients

**DOI:** 10.1515/med-2022-0585

**Published:** 2022-10-31

**Authors:** Li-ping Su, Min Ji, Li Liu, Wei Sang, Jing Xue, Bo Wang, Hong-Wei Pu, Wei Zhang

**Affiliations:** Department of Science and Research Education Center, The First Affiliated Hospital, Xinjiang Medical University, No. 137 Liyushan Southern Road, Urumqi, Xinjiang 830011, P.R. China; Xinjiang Medical University, Urumqi, Xinjiang 830011, P.R. China; Department of Pathology, The First Affiliated Hospital, Xinjiang Medical University, Urumqi, Xinjiang 830011, P.R. China; Department of Pathology, The First Affiliated Hospital, Xinjiang Medical University, State Key Laboratory of Pathogenesis, Prevention and Treatment of High Incidence Diseases in Central Asia, Urumqi, Xinjiang 830011, P.R. China; College of Basic Medicine, Xinjiang Medical University, Urumqi, Xinjiang 830011, P.R. China; Department of Pathology, The First Affiliated Hospital, Xinjiang Medical University, No. 137 Liyushan Southern Road, Urumqi, Xinjiang 830011, P.R. China

**Keywords:** glioma, bioinformatics, clinicopathologic characteristics, ASAP3, NOTCH3

## Abstract

*ASAP3* is involved in a variety of biological activities, including cancer progression in humans. In adult glioma, we explore the effects of *ASAP3* and *NOTCH3* and their relationships on prognosis. The Oncomine, TIMER, and Gene Expression Profiling Interactive Analysis databases were used to investigate *ASAP3* expression. Immunohistochemistry was used to assess the levels of *ASAP3* and *NOTCH3* expressions. The effects of *ASAP3* and *NOTCH3* on prognosis were assessed using survival analysis. The results revealed that the amount of *ASAP3* mRNA in gliomas was much higher than in normal tissue (*P* < 0.01). Glioma patients with high *ASAP3* mRNA expression had a worse overall survival and progression-free survival. *ASAP3* overexpression is directly associated with the NOTCH signaling system. Immunohistochemistry revealed that *ASAP3* and *NOTCH3* were overexpressed in glioblastomas (GBMs). *ASAP3* expression was associated with age, recurrence, tumor resection, postoperative chemoradiotherapy, World Health Organization (WHO) grade, and *Ki-67* expression. *ASAP3* expression was related to the isocitrate dehydrogenase-1 mutation in low-grade glioma. Gender, local recurrence, tumor resection, postoperative radio-chemotherapy, WHO grade, recurrence, and *ATRX* expression were all associated with *NOTCH3* expression. *ASAP3* was shown to be positively associated with *NOTCH3* (*r* = 0.337, *P* = 0.000). Therefore, *ASAP3* and *NOTCH3* as oncogene factors have the potential to be prognostic biomarkers and therapeutic targets in adult glioma.

## 1 Introduction

Adult diffuse glioma is the most prevalent malignant brain tumor, accounting for over 80% of all malignant brain and central nervous system (CNS) cancers. Glioma is an aggressive and lethal solid tumor generated by glial cells and among the most prevalent malignant tumors in the brain [1]. According to epidemiological statistics, glioblastomas (GBMs) are malignant tumors, which account for 30% of CNS tumors and 80% of malignant brain tumors in the whole world [2]. Glioma is a refractory solid tumor that is resistant to chemo and radiotherapy. Although combination therapy has improved the prognosis of adult glioma, the prognosis of adult glioma remains dismal, with a median survival of 14–16 months. Following the World Health Organization's (WHO) reclassification of CNS tumors in 2021, a new diagnostic concept was adopted, combining tumor histology and molecular genetics, such as isocitrate dehydrogenase (IDH) mutations and 1P/19Q co-deletion states, TRET, Alpha Thalassemia/Mental Retardation Syndrome X-Linked *(ATRX)*, and morphologic changes, to form a more accurate diagnosis [3]. Patients who were graded as 2–3 low-grade glioma (LGG) survived for more than 10 years before developing highly invasive grade glioma due to aberrant activation of several oncogenes and signaling pathways [4]. With the advancement of genomics and bioinformatics databases, the cancer genome atlas (TCGA), Oncomine, and TIMER data sites have been promoted to deeply understand the genomes of glioma occurrence and progression, and targeted gene therapy is emerging as a promising glioma-accurate drug treatment strategy. However, the intricacy of the signaling pathways involved in gliomagenesis raises confusion regarding the optimal target and limits the utility of targeted treatment in glioma. The processes that cause glioma cell invasion are mostly unknown. Further understanding the mechanism of the malignant invasion and molecular targeted therapy of glioma can lay a theoretical foundation for finding effective biomarkers and potential carcinogenic pathways to fight against this invasive tumor.

*ASAP3*, also known as *ACAP4*, is a GTPase-activating protein (GAP) for the ADP-ribosylation factor 6 (ARF6) and possesses the BAR, PH, ankyrin repeat, and GAP domains. Okabe et al. initially identified it as a development and differentiation enhancing factor (called *DDEFL1*) and demonstrated that it promoted the proliferation of hepatocellular carcinoma cells [5]. Given that *DDEFL1* and ACAPs family proteins share a similar domain structure organization and substrate, *DDEFL1* was renamed ACAP4 and found to be a particular GAP protein for ARF6 and a critical participant in cell migration [6]. *ASAP3* expression is minimal or nonexistent in normal epithelia, but it has been reported to be significantly increased in a variety of human carcinomas, including lung carcinomas, colon cancers, and breast cancers, and *ASAP3* expression may contribute to a poor clinical outcome in non-small cell lung carcinoma and colon cancer [7,8,9]. These effects may be attributable to the role of *ASAP3* in regulating cell migration and, by extension, cancer cell invasion. Epidermal growth factor (EGF) activation causes EGF receptor (EGFR) kinase to phosphorylate *ASAP3* at Tyr34, and CCL18 therapy causes *ASAP3* Lys311 to be acetylated. These post-translational changes play a vital role in regulating the localization of *ASAP3* at focal adhesions during cell migration [10]. However, the molecular processes underlying *ASAP3* overexpression and the role of *ASAP3* in glioma progression remain largely unknown. This study is intended to evaluate the potential biological function of *ASAP3* in the progression of adult glioma.

The NOTCH signaling pathway is an evolutionarily conserved pathway that is crucial for both normal embryonic development and malignancy. The pathway is also frequently implicated in neoplasia and promotes neoplastic growth in most situations [11]. Notch3 is a component of the signaling cascade, including NOTCH ligands, NOTCH receptors, and transcription factors. The oncogenic function of *NOTCH3* has been documented in esophageal cancer [12], ovarian cancer [13], hepatocellular carcinoma [14], and so on. Furthermore, it has been proven that cross-regulation between *EGFR* and *NOTCH3* has long been detected in genetic studies and that, depending on the cellular context, it can be both cooperative and antagonistic [15]. *NOTCH3* promotes glioma cell invasion and proliferation through activation of cell cycle protein D1 (*CCND1*) and *EGFR* gene expression. Studies have shown that *NOTCH3* gene polymorphisms have the potential to be diagnostic and therapeutic biomarkers for gliomas.

Our team has been investigating the glioma molecular mechanism. In our early studies, Ingenuity Pathway Analysis was used to confirm that Notch signaling was activated in GBM with a *Z* score of 1.342 and *P*-value of 0.029, and that *ASAP3* was activated with a fold change of 1.54 and a *P*-value of 0.0009. Gene Expression Profiling Interactive Analysis (GEPIA) was used to validate the expression of *ASAP3* as well as the correlation of NOTCH signaling pathway proteins in gliomas. Although it has been reported that the expression of *ASAP3* is elevated in glioma, its prognostic value and relationship with the expression of the cooperative protein *NOTCH3* remain unclear. We intend to investigate the biological function of *ASAP3* and its closely related protein *NOTCH3*, as well as provide new hints for molecularly targeted glioma therapy, by elucidating the expression and clinical significance of *ASAP3* in adult glioma.

## 2 Materials and methods

### 2.1 Analysis of the expression of *ASAP3* in tumors and normal tissues

#### 2.1.1 Tissues in human cancers

The Oncomine database (https://www.oncomine.org/resource/login.html), the TIMER database (https://cistrome.shinyapps.io/timer/), and the GEPIA database (http://gepia2.cancer-pku.cn/#analysis) were utilized to evaluate *ASAP3* expression between human tumor and paired normal tissue. The data on tumors and normal tissues are analyzed by the GEPIA database, which is a website. This database looks at the data from the TCGA database.

### 2.2 The prognostic relevance of *ASAP3* in glioma

Using the GEPIA database (http://gepia2.cancer-pku.cn/#analysis), the prognostic significance of *ASAP3* expression in glioma was investigated. We examined the relationship between *ASAP3* expression and overall survival (OS) and disease-free survival (DFS) in glioma using the GEPIA database. In the GEPIA database, the median *ASAP3* expression was applied to classify groups as the cutoff value.

### 2.3 GSEA identifies *ASAP3*-associated signaling pathways in glioma

The glioma data, mRNA expression profiles, and survival information for 169 glioma patients were downloaded from the TCGA Genomic Data Commons data portal (https://portal.gc.cancer.gov/repository). GSEA is a computer program that determines if a priori-defined collection of genes exhibits statistically significant differential expression between high expression and low expression groups. Generated datasets and phenotype label files were submitted to the GSEA software. The phenotypes were labeled as *ASAP3*-high and *ASAP3*-low. For each analysis, 1,000 permutations of the gene set were performed. Generally regarded as enriched were gene sets with a normal *P*-value <0.05 and a false discovery rate <0.25.

### 2.4 Analysis of *ASAP3*’s interaction network with the NOTCH signaling pathway

The GEPIA database was used to generate a scatter plot and related genes. The GeneMANIA database was used to analyze protein–protein interactions, genetic pathways, and protein co-expression networks in the *ASAP3* gene with the NOTCH signaling pathway.

### 2.5 The function of the *ASAP3* gene

WebGestalt is a website that analyses gene function enrichment. WebGestalt analyzed the key genes of the NOTCH signaling pathway that interact with *ASAP3* to investigate the involved cell components, biological processes, and biological functions.

### 2.6 Immunohistochemistry and assessment of the intensity of immunostaining

The Department of Pathology at the First Affiliated Hospital of Xinjiang Medical University constructed tissue microarrays (TMAs), while Shanghai Outdo Biotech Company provided technical support (Shanghai, China). The TMA was built from formalin-fixed paraffin-embedded blocks of 211 adult glioma surgical resections, which were reviewed in each case to confirm the original diagnosis and select the most representative sections using Hematoxylin and Eosin (HE) stained slides. All the surgical resections came from the First Affiliated Hospital of Xinjiang Medical University between July 2010 and November 2020. All glioma biopsies were reviewed by two professional pathologists, and any inconsistent diagnosis was further reviewed by a third professional pathologist in order to reach a final determination.

The gliomas of grades 2 and 3 were classified as LGG, whereas the glioblastoma was classified as GBM. Among all the 211 cases of gliomas, 138 were LGG and 73 were GBM. The detection kit was used for immunostaining (ZSGB-BIO, SP-9001, China). Anti-*ASAP3* (SC-365840, 1:100, Santa) and anti-NOTCH3 (ab23426, 1:100, Abcam) antibodies were incubated overnight at 4°C on the sections. All slides were dehydrated and counterstained with diaminobenzidine solution (ZSGB-BIO, ZLI-9017, China) for 2 min and hematoxylin (Solarbio, G1140, China). The intensity of the IHC staining was graded as 0 (no), 1 (weak), 2 (medium), and 3 (strong). The staining extent was graded from 0 to 3 based on the percentage of immune-reactive tumor cells (0–10%, 21–75%, and 76–100%). For each example, a score ranging from 0 to 9 was calculated by multiplying the staining extent score by the staining intensity score, resulting in low (0–4) or high (5–9) staining. *ASAP3* staining expresses cytoplasm. *NOTCH3* staining revealed the cell membrane and nucleus.


**Ethics approval and consent to participate:** This study was approved by the ethics committee of the First Affiliated Hospital of Xinjiang Medical University. A Signed written informed consent was obtained from all participants before the study.

### 2.7 Statistical methods

Statistical analysis was performed using SPSS 26.0 Software (SPSS Inc., Chicago, IL, USA) and GraphPad Prism 8.0 Software (GraphPad Software Inc., San Diego, CA, USA). The *χ* ^2^ test and Pearson correlation coefficient were used to examine the expression of *ASAP3* and *NOTCH3* in adult gliomas. The OS and PFS were analyzed by the Kaplan–Meier method and the log-rank method. The cox proportional risk regression model was used for multivariate analysis. Statistical analysis results with *P* < 0.05 were considered statistically significant, offering credibility for the above data analysis.

## 3 Results

### 3.1 *ASAP3* expression levels in different types of human cancers

**Figure 1:**
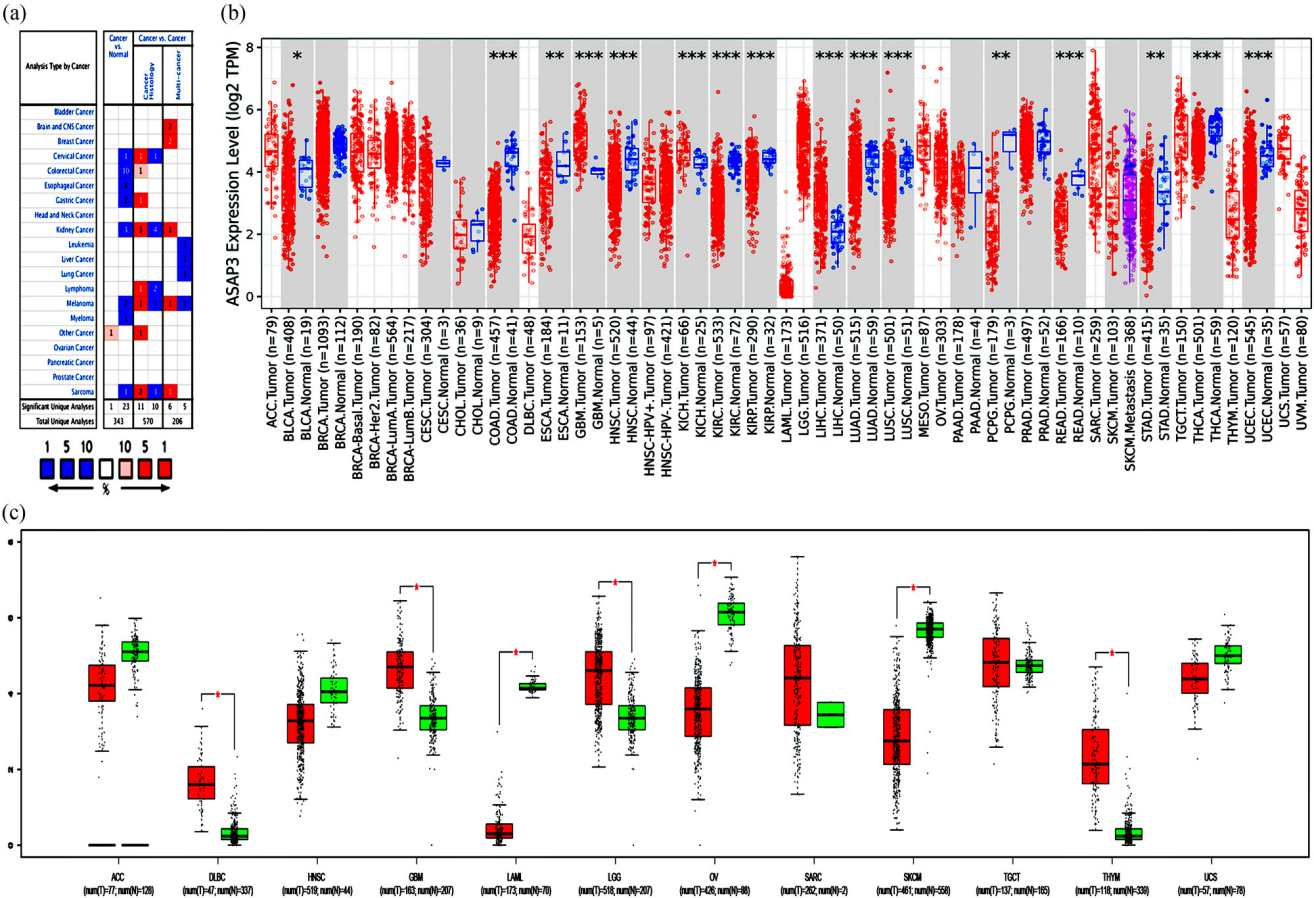
ASAP3 expression levels in different types of human cancer. (a) The expression of ASAP3 in different cancers by the Oncomine database; the P-value threshold is set to 0.05, and the fold change requirement is set to 2. (b) The expression of ASAP3 in different cancer types by the TIMER database; *P < 0.05, **P < 0.01, ***P < 0.001. (c) The expression of ASAP3 in several cancers and paired normal tissue by the GEPIA database; *P < 0.05.

We determined the expression difference of *ASAP3* between tumor tissues and normal tissues through multiple databases. According to the Oncomine database, *ASAP3* expression was higher in cervical cancer, colorectal cancer, gastric cancer, kidney cancer, melanoma, and lymphoma tumors in cancer histology. It is also higher in multicancer with Brain and CNS cancer, kidney, melanoma, and breast cancer in some datasets (Figure 1a). The results of the TIMER database analysis showed that *ASAP3* expression was significantly higher in GBM (Glioblastoma multiforme), kidney chromophobe (KICH), and liver hepatocellular carcinoma (LIHC) compared with adjacent normal tissues. However, *ASAP3* expression was significantly lower in bladder urothelial carcinoma (BLCA), colon adenocarcinoma (COAD), esophageal carcinoma (ESCA), head and neck cancer (HNSC), kidney renal clear cell carcinoma (KIRC), lung adenocarcinoma (LUAD), Kidney renal papillary cell carcinoma (KIRP), lung squamous cell carcinoma (LUSC), Pheochromocytoma and Paraganglioma (PCPG), rectum adenocarcinoma (READ), stomach adenocarcinoma (STAD), thyroid carcinoma (THCA), and uterine corpus endometrial carcinoma (UCEC) when compared with adjacent normal tissues (Figure 1b). The results of the GEPIA database analysis were used as supplementary results for cancers without paired normal tissues in the TIMER database. Meanwhile, the results showed that the expression of *ASAP3* mRNA was also significantly higher in other cancer types: Lymphoid Neoplasm Diffuse Large B-Cell Lymphoma (DLBC), Brain LGG, and Thymoma (THYM) (Figure 1c). These results suggest that the expression levels of ASAP3 are inconsistent in different tumor tissues, which may be related to the different pathogenesis processes of tumors. However, *ASAP3* may be involved in the process of glioma regulation.

### 3.2 *ASAP3* is a prognostic glioma biomarker

**Figure 2:**
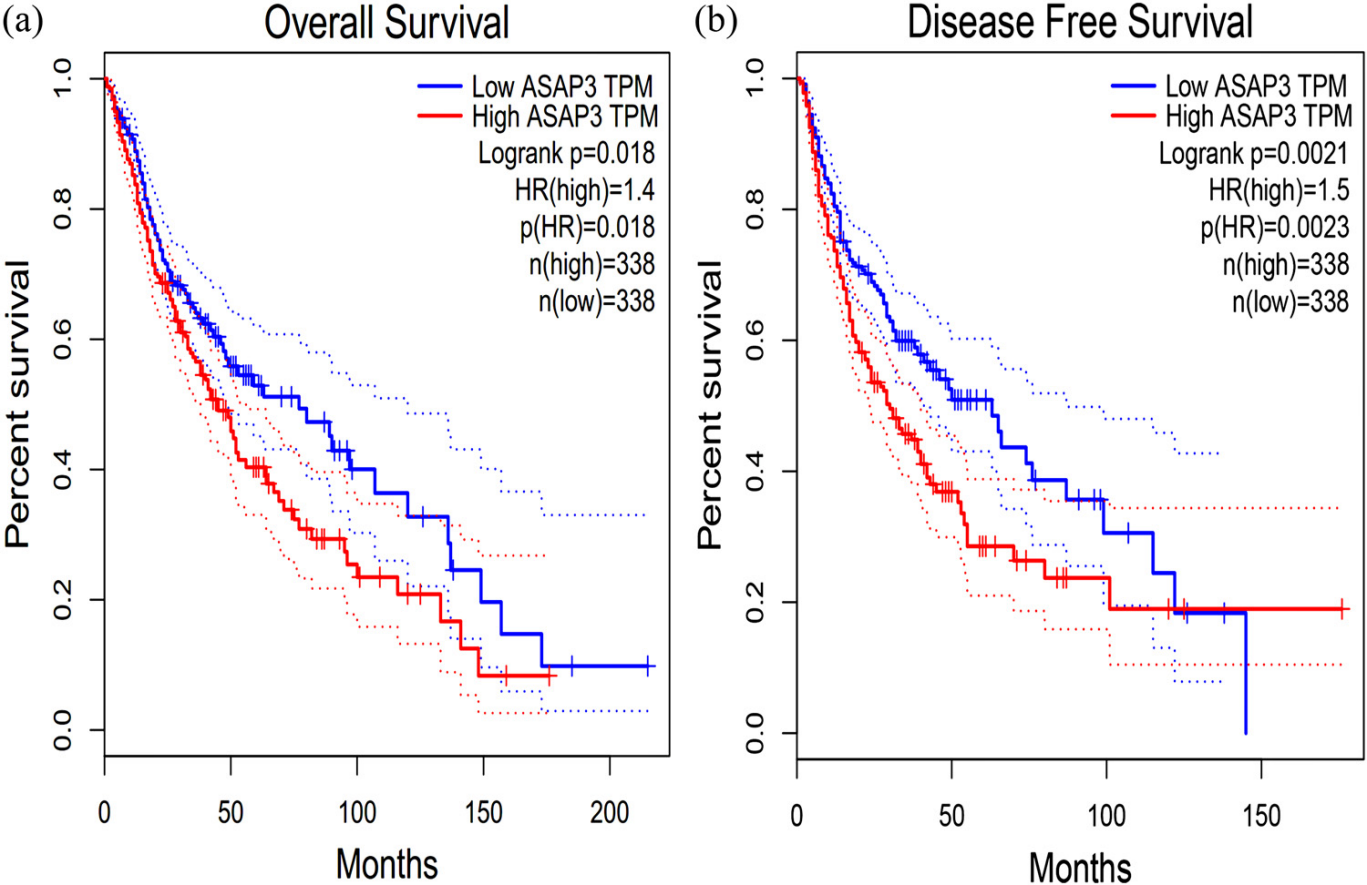
Analysis of the expression of ASAP3 in the survival of glioma patients. (a) The expression of ASAP3 in the OS of glioma patients. (b) The expression of ASAP3 in the DFS of glioma patients.

We used the GEPIA database to analyze the prognostic value of *ASAP3* expression in glioma. We analyzed the correlation between *ASAP3* expression levels and the OS and PFS of 676 glioma patients. According to the median expression, the 676 glioma patients were split into the *ASAP3* high expression group (*n* = 338) and the *ASAP3* low expression group (*n* = 338). These results of the GEPIA database showed that higher *ASAP3* expression was associated with OS and DFS in gliomas (*n* = 338, OS: Hazard Ratio (HR) = 1.4, *P* = 0.018; *n* = 338, DFS: HR = 1.5, *P* = 0.0023). This was determined by HR, which is a statistical measure of the likelihood of an event occurring. There was a statistical significance to the results (Figure 2a and b).

### 3.3 GSEA identified signaling pathways associated with *ASAP3*

**Figure 3:**
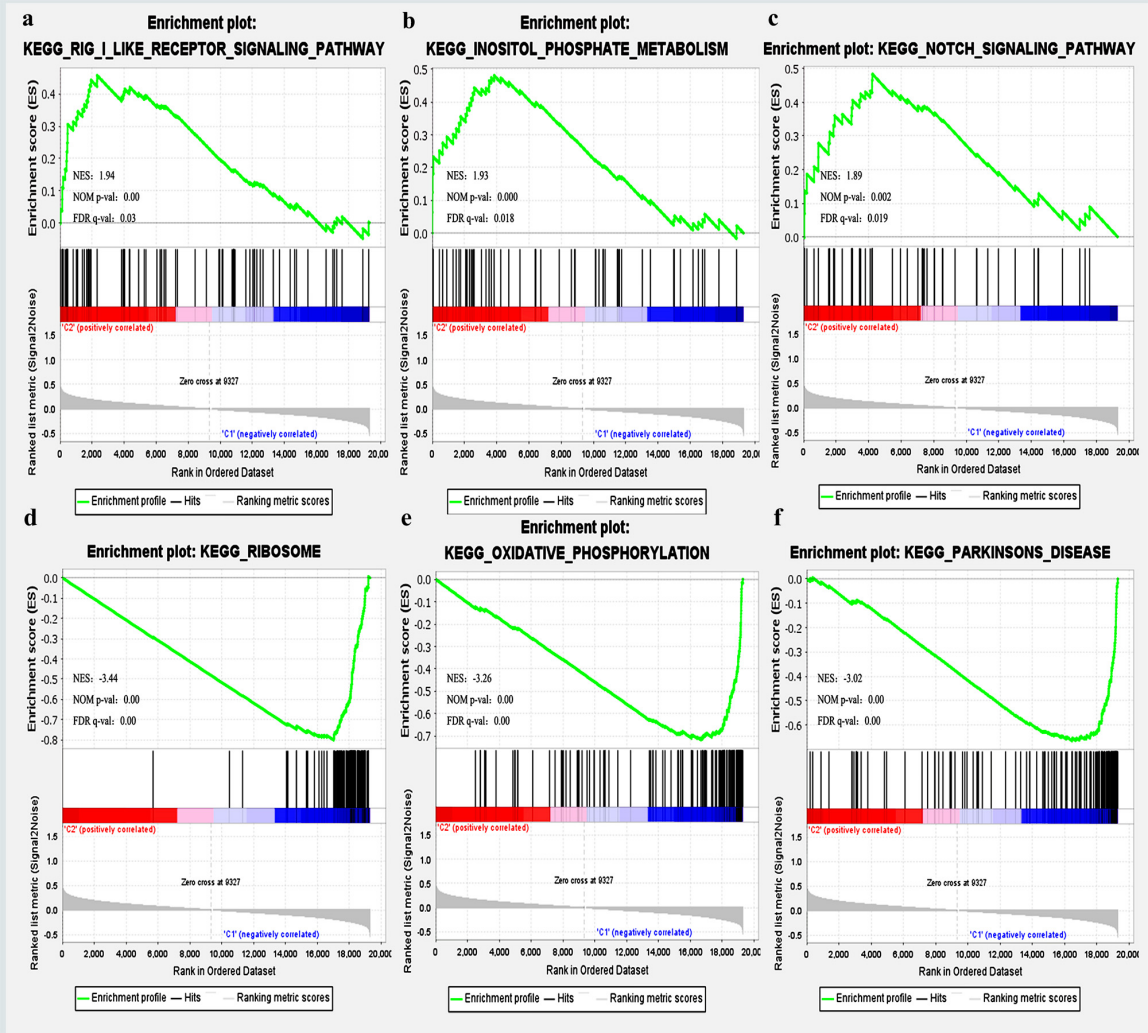
Potential downstream signal pathways of ASAP3 are revealed through GSEA analysis. Based on GSEA data results, Rig like receptor signaling pathway (a), Inositol phosphate metabolism (b), and NOTCH signaling pathway (c) were enriched in the high ASAP3 expression group. Ribosome (d), oxidative phosphorylation (e), and Parkinson's disease (f) were enriched in the low ASAP3 expression group. The scores for each gene's enrichment are displayed in the top panels. The lower panels display the ranking metrics for each gene. *X*-axis: rankings for all genes; *Y*-axis: values of ranking metrics. NES stands for normalized enrichment score.

On the basis of the TCGA-STAD dataset, we used GSEA to identify biological pathways that may be influenced by *ASAP3* in the tumors. Using GSEA (h.all.v6.2.symbols.gmt), significant differences in the enrichment of the MSigDB dataset were discovered. The analysis result displayed that the *ASAP3* high expression phenotype was associated with the Rig like receptor signaling pathway (Figure 3a), Inositol phosphate metabolism (Figure 3b), NOTCH signaling pathway (Figure 3c), Adherens junction, Alpha linolenic acid metabolism, Phosphatidylinositol signaling system, ABC transporters, Acute myeloid leukemia, Basal cell carcinoma, Pancreatic cancer, etc. The Ribosome (Figure 3d), Oxidative Phosphorylation (Figure 3e), Parkinson disease (Figure 3f), Alzheimer’s disease, Huntington disease, and Proteasome were differentially associated with the *ASAP3* low expression phenotype (Table 1).

**Table 1:** Results of GSEA analysis based on the expression level of *ASAP3* gene

Group	Term	ES	NES	NOM *P*-val
High ASAP3	KEGG_RIG_I_LIKE_RECEPTOR_SIGNALING_PATHWAY	0.46	1.94	0.000
	KEGG_INOSITOL_PHOSPHATE_METABOLISM	0.48	1.93	0.000
	KEGG_NOTCH_SIGNALING_PATHWAY	0.48	1.89	0.002
	KEGG_ADHERENS_JUNCTION	0.44	1.84	0.000
	KEGG_ALPHA_LINOLENIC_ACID_METABOLISM	0.58	1.79	0.006
	KEGG_PHOSPHATIDYLINOSITOL_SIGNALING_SYSTEM	0.4	1.71	0.000
	KEGG_ABC_TRANSPORTERS	0.44	1.66	0.004
	KEGG_ACUTE_MYELOID_LEUKEMIA	0.41	1.66	0.006
	KEGG_BASAL_CELL_CARCINOMA	0.41	1.64	0.002
	KEGG_PANCREATIC_CANCER	0.39	1.62	0.009
Low ASAP3	KEGG_RIBOSOME	−0.8	−3.44	0.000
	KEGG_OXIDATIVE_PHOSPHORYLATION	−0.72	−3.26	0.000
	KEGG_PARKINSON’S_DISEASE	−0.66	−3.02	0.000
	KEGG_ALZHEIMER’S_DISEASE	−0.58	−2.73	0.000
	KEGG_HUNTINGTON_DISEASE	−0.57	−2.73	0.000
	KEGG_PROTEASOME	−0.69	−2.59	0.000
	KEGG_CARDIAC_MUSCLE_CONTRACTION	−0.56	−2.34	0.000
	KEGG_OOCYTE_MEIOSIS	−0.43	−1.92	0.000
	KEGG_DNA_REPLICATION	−0.52	−1.9	0.000
	KEGG_CELL_CYCLE	−0.41	−1.84	0.000

### 3.4 Prediction and functional analysis of the *ASAP3* protein interaction NOTCH signaling pathway network and GO analysis

**Figure 4:**
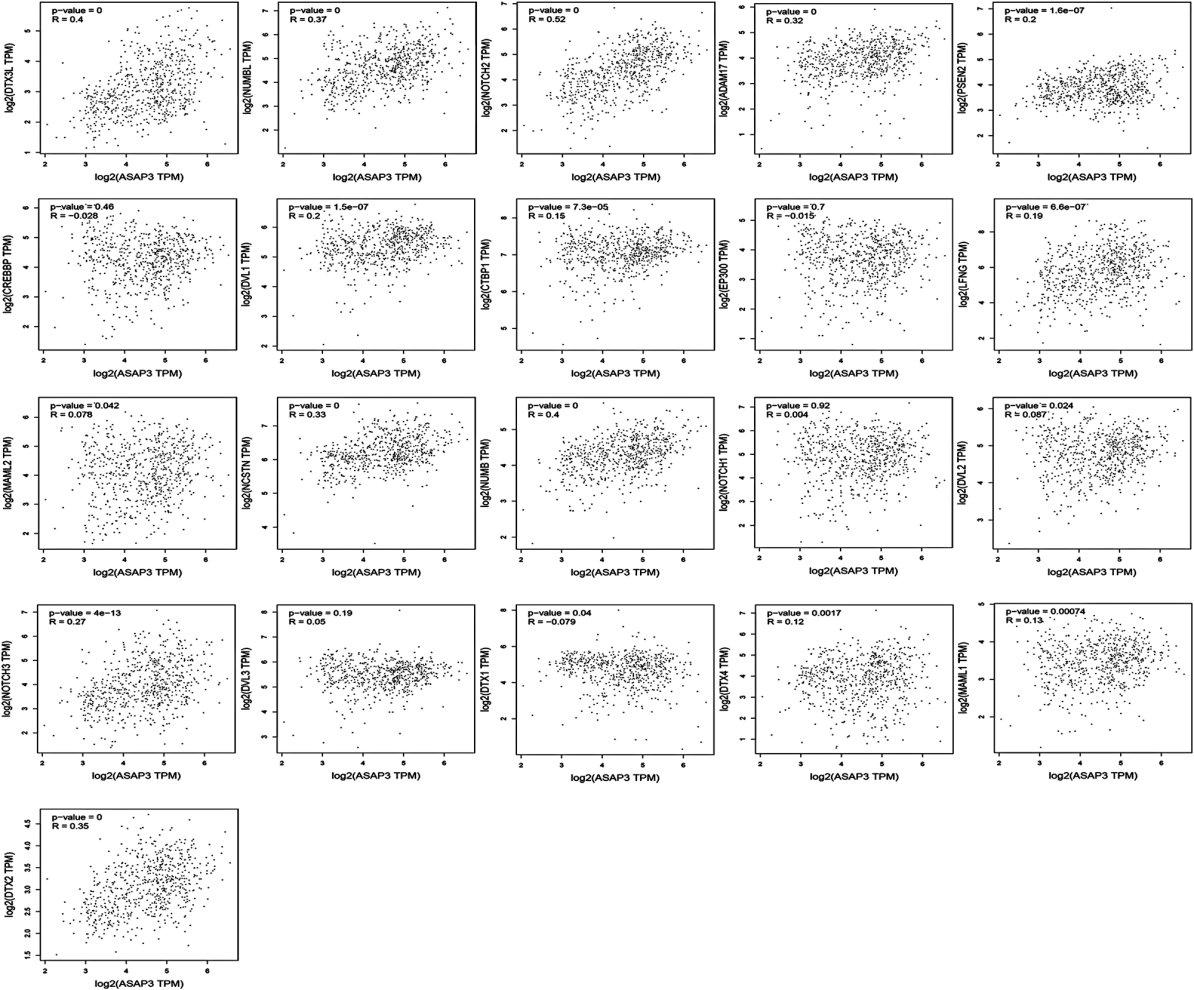
Analysis of the correlation between ASAP3 and each gene in the NOTCH signaling pathway.

**Figure 5:**
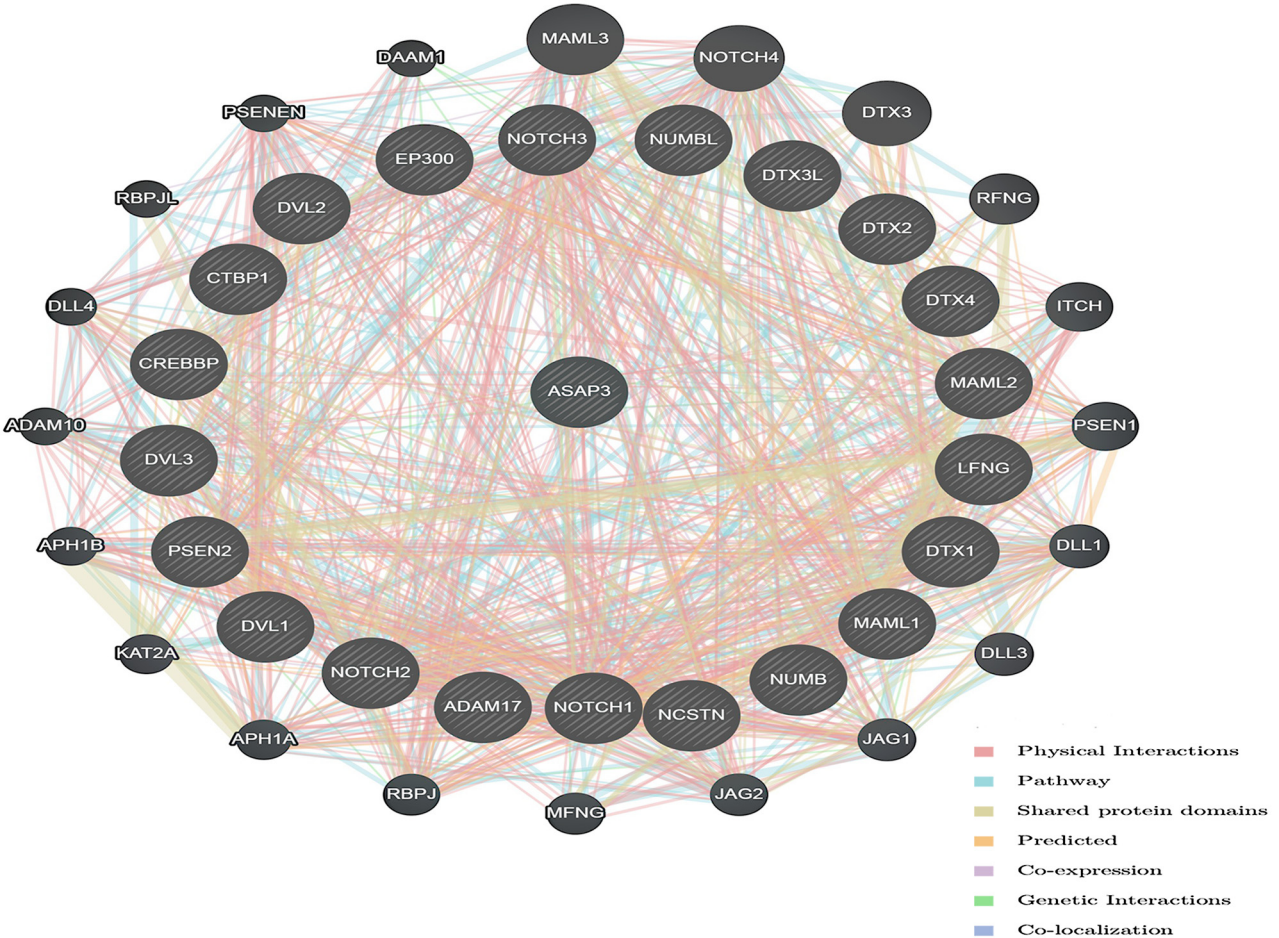
Analysis of ASAP3-interacting proteins by GeneMANIA.

**Figure 6:**
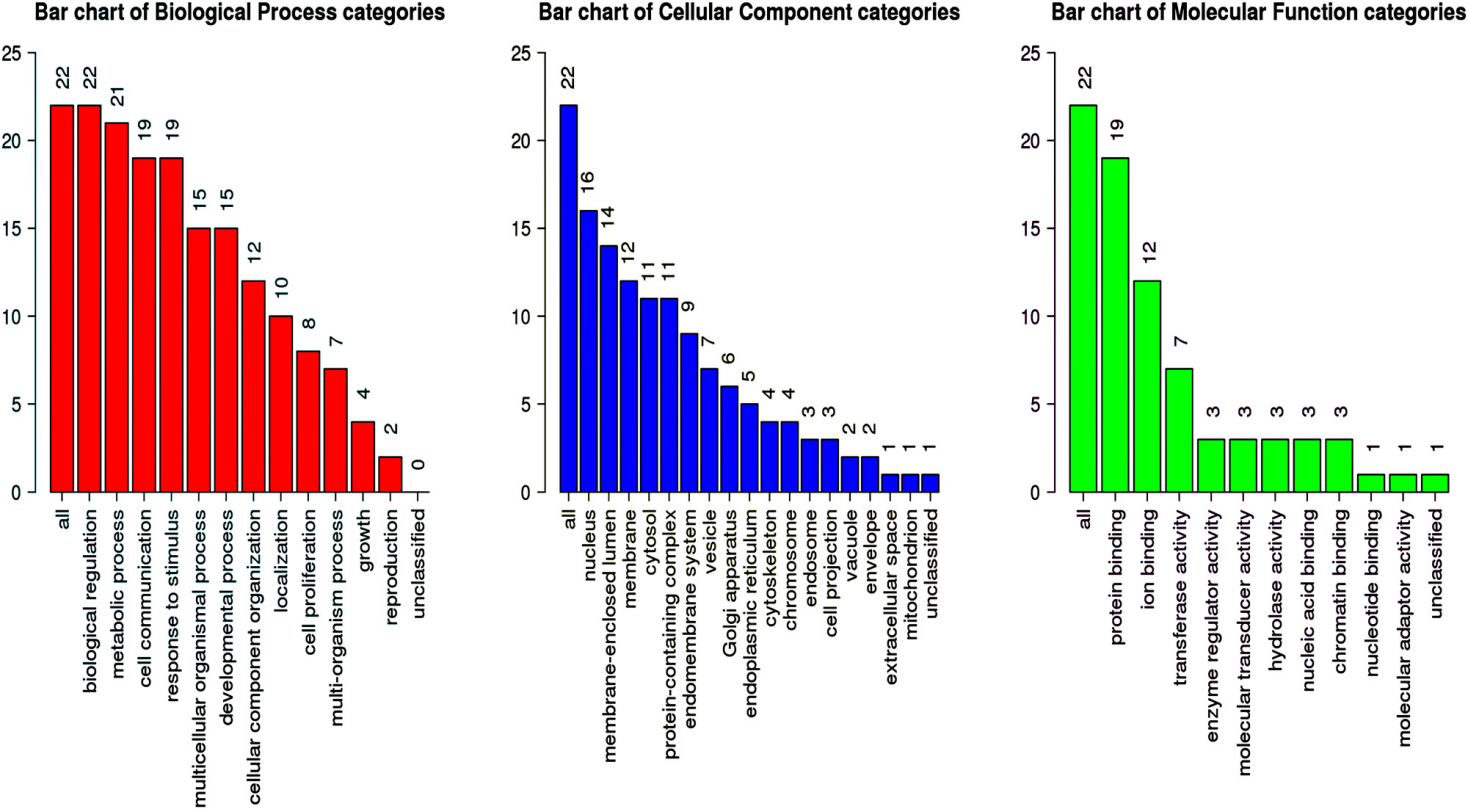
Analysis of ASAP3-related biological processes, cell components, and molecular activities.

Using GeneMANIA analysis, we screened a total of 21 genes interacting with *ASAP3* (The core enrichment of GSEA was yes in the NOTCH signaling pathway): *DTX3L, NUMBL, NOTCH2, ADAM17, PSEN2, CREBBP, DVL1, EP300, CTBP1, LFNG, MAML2, NCSTN, NUMB, NOTCH1, DVL2, NOTCH3, DVL3, DTX1, DTX4, MAML1*, and *DTX2*. We focused on analyzing the interaction relationship between *ASAP3* and NOTCH signaling pathway proteins, where the main components are physical interactions, co-expression, shared protein domains, and co-localization. The results showed the correlation scatter plots for *ASAP3* and each gene individually in Figure 4. The interaction network showed that these proteins were found to be mainly engaged in biological regulation, metabolic processes, cellular communication, etc. (Figure 5). Genes associated with *ASAP3* may be found in many different parts of the cell, such as the nucleus, membrane-enclosed lumens, and membranes. Among the several molecular roles are protein binding, ion binding, and transferase activity (Figure 6).

### 3.5 Clinicopathologic characteristics of 211 cases of adult glioma

The clinicopathologic characteristics of 211 patients with adult glioma are shown in supplement Table A1. We mainly analyzed the composition of LGG (65.4%; 138/211) and GBM (34.6%; 73/211) in this cohort. The overall cohort's clinical information included tumor size ≥ 3 cm (82.46%; 174/211), total tumor resection (67.3%; 142/211), and no local recurrence (65.4%; 138/211). There were significant differences in age, tumor location, local recurrence, tumor resection, Ki-67 expression, and p53 expression between LGG and GBM patients.

### 3.6 The protein expression of *ASAP3* and NOTCH3 in adult glioma and their relationship with clinicopathological parameters

**Figure 7:**
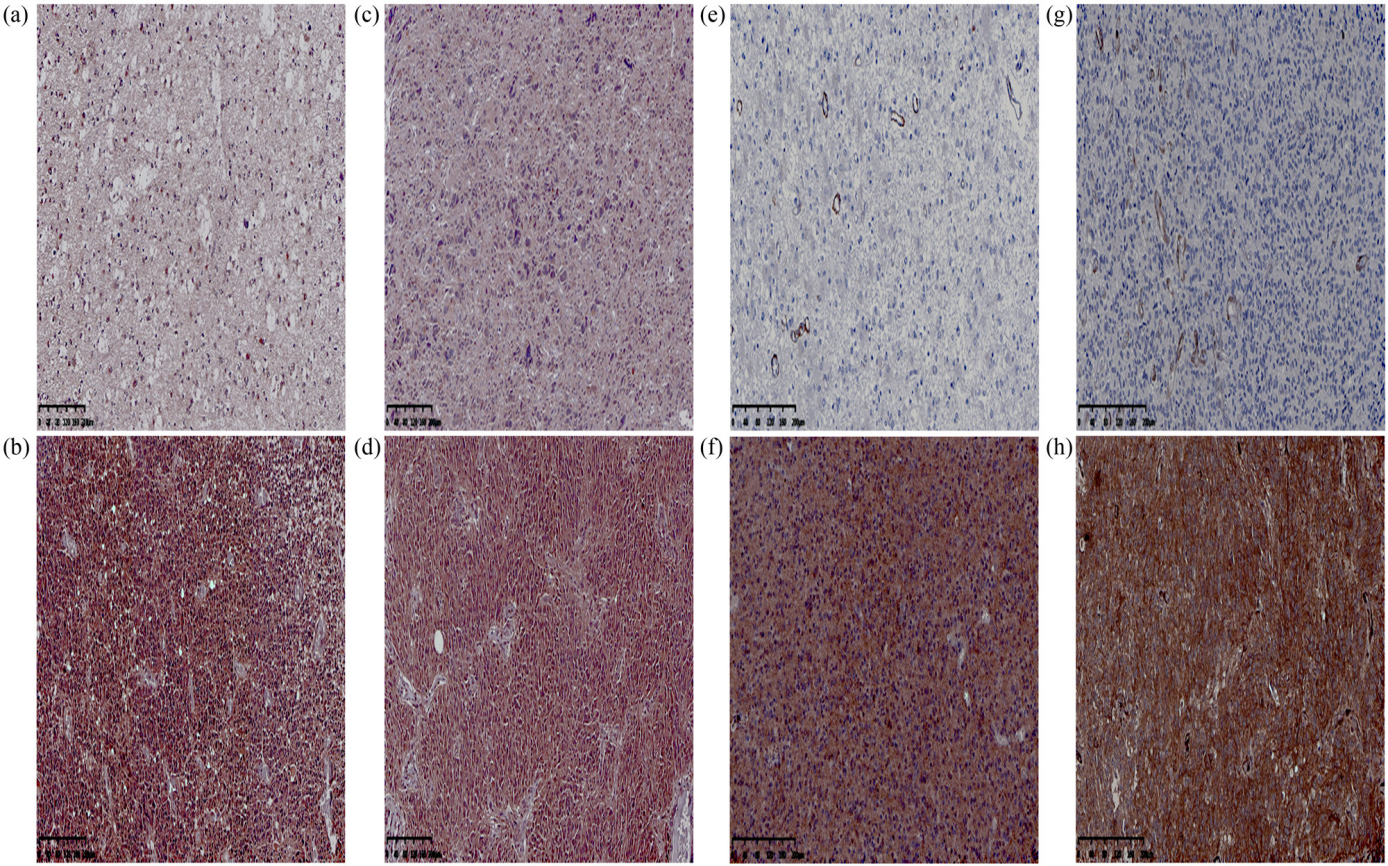
The expressions of ASAP3 and NOTCH3 were detected by immunohistochemistry. (a) Low expression of ASAP3 in LGG. (b) High expression of ASAP3 in LGG. (c) Low expression of ASAP3 in GBM. (d) High expression of ASAP3 in GBM. (e) Low expression of NOTCH3 in LGG. (f) High expression of NOTCH3 in LGG. (g) Low expression of NOTCH3 in GBM. (h) High expression of NOTCH3 in GBM. Magnification ×200.

Immunohistochemistry was used to detect the protein expression of *ASAP3*, *NOTCH3*, and their relationship with clinicopathological features in all cohorts in order to study the potential function of *ASAP3* and *NOTCH3* in the progression of adult glioma (Figure 7). In this cohort, the expression of *ASAP3* was found to be high in 123 tumors (58.29%), whereas it was low in 88 tumors (41.71%). The high expression of *ASAP3* in GBM (80.82%) suggested that *ASAP3* is activated more frequently in the most aggressive and malignant tumors (in Table 2). The results of the statistical analysis showed that *ASAP3* expression was related to age, recurrence, tumor resection, postoperative radio-chemotherapy, WHO grade, and *Ki-67* expression; however, its expression was not related to gender, ethnicity, tumor size, tumor location, *p53* expression, and *ATRX* expression (Table 3). Meanwhile, the relationship between *ASAP3* and 1p19q codeletion in LGG had no statistical significance (*P* = 0.798). We analyzed the IDH1 mutation status of LGG and found 113 cases of *IDH1* mutation (termed IDH1mut) and 25 cases of IDH1 wild type (termed IDH1wt). We detected high *ASAP3* expression in 40.71% of IDH1mut (46/113) and 72.00% of IDH1wt (18/25), indicating that *ASAP3* was expressed significantly differently between IDH1mut and IDH1wt (*P* = 0.004). Spearman results revealed the negative relationship between IDH1 mutation and *ASAP3* expression (*P* = 0.004, *r* = −0.242) (Table 4).

**Table 2:** Staining results of ASAP3 and NOTCH3 on the adult glioma specimens (*n* = 211)

Antibody	Grade	Low expression	High expression	*P* value
ASAP3 [*n* (%)]	LGG	74 (74/138, 53.62%)	64 (64/138, 46.38%)	<0.0001
	GBM	14 (14/73, 19.18%)	59 (59/73, 80.82%)	
NOTCH3 [*n* (%)]	LGG	83 (83/138, 60.14%)	55 (55/138, 39.86%)	<0.0001
	GBM	19 (19/73, 26.03%)	54 (58/73, 73.97%)	

*P* values were calculated by Pearson’s *χ* ^2^ test (two sided). *P* < 0.05 indicates statistical significance. LGG, low-grade gliomas; GBM, glioblastoma.

**Table 3:** Clinicopathological characteristics according to protein expressions of ASAP3 and NOTCH3 in all glioma patients (*n* = 211)

Clinicopathologic characteristics	ASAP3 expression [*n* (%)]			NOTCH3 expression [*n* (%)]		
	Low	High	*P*-value	Low	High	*P*-value
**Age**			0.030*			0.070
≤50	59 (50.9%)	57 (49.1%)		55 (47.4%)	61 (52.6%)	
>50	29 (30.5%)	66 (69.5%)		33 (34.7%)	62 (65.3%)	
**Gender**			0.672			0.048*
Male	53 (43.1%)	70 (56.9%)		44 (35.8%)	79 (64.2%)	
Female	35 (39.8%)	53 (60.2%)		44 (50%)	44 (50%)	
**Ethnic**			0.674			0.532
Han	51 (43.2%)	67 (56.8%)		49 (41.5%)	69 (58.5%)	
Other	37 (39.8%)	56 (60.2%)		39 (41.9%)	54 (58.1%)	
**Size of the main lesion**			0.513			0.364
<3 cm	15 (40.5%)	22 (59.5%)		18 (48.6%)	19 (51.4%)	
≥3 cm	73 (42%)	101 (58%)		70 (40.2%)	104 (59.8%)	
**Tumor location**			0.485			0.889
Frontal	48 (44.4%)	60 (55.6%)		46 (42.6%)	62 (57.4%)	
Other	40 (38.8%)	63 (61.2%)		42 (40.8%)	61 (59.2%)	
**Local recurrence**			0.000*			0.000*
No	72 (52.2%)	66 (47.8%)		76 (55.1%)	62 (44.9%)	
Yes	16 (21.9%)	57 (78.1%)		12 (16.4%)	61 (83.6%)	
**Tumor resection**			0.002*			0.025*
Total resection	70 (49.3%)	72 (50.7%)		67 (47.2%)	75 (52.8%)	
Partial resection	18 (26.1%)	51 (73.9%)		21 (30.4%)	48 (69.6%)	
**Postoperative radio-chemotherapy**			0.026*			0.012*
No	56 (48.7%)	59 (51.3%)		57 (49.6%)	58 (50.4%)	
Yes	32 (33.3%)	64 (66.7%)		31 (32.3%)	65 (67.7%)	
**WHO grade**			0.000*			0.000*
II	60 (65.9%)	31 (34.1%)		56 (61.5%)	35 (38.5%)	
III	14 (29.8%)	33 (70.2%)		17 (36.2%)	30 (63.8%)	
IV	14 (19.2%)	59 (80.8%)		15 (20.5%)	58 (79.5%)	
**Ki-67 expression**			0.002*			0.051
≤5%	55 (52.4%)	50 (47.6%)		51 (48.6%)	54 (51.4%)	
>5%	33 (31.1%)	73 (68.9%)		37 (34.9%)	69 (65.1%)	
**P53 expression**			0.513			0.516
≤5%	53 (41.4%)	75 (58.6%)		50 (39.1%)	78 (60.9%)	
>5%	35 (42.2%)	48 (57.8%)		33 (39.8%)	50 (60.2%)	
**ATRX expression**			0.073			0.003*
wt	27 (52.9%)	24 (47.1%)		30 (58.8%)	21 (41.2%)	
mut	61 (38.1%)	99 (61.9%)		56 (35%)	104 (65%)	

Statistical analyses were performed by the Pearson *χ* ^2^ test (two sided). *P < 0.05 was considered statistically significant.

**Table 4:** The expression of ASAP3 and NOTCH3 in LGG according to the IDH1 gene and 1p19q state

Molecular	Profiles	ASAP3		*r*	*P*-value	NOTCH3		*r*	*P*-value
		Low	High			Low	High		
IDH1	wt	7 (28.00%)	18 (72.00%)	−0.242	0.004	11 (44.00%)	14 (56.00%)	−0.084	0.328
	mut	67 (59.29%)	46 (40.71%)			62 (54.87%)	51 (45.13%)		
1p9q	codel	27 (55.10%)	22 (44.90%)	0.022	0.798	29 (59.18%)	20 (40.82%)	0.093	0.276
	non-codel	47 (52.81%)	42 (47.19%)			44 (49.44%)	45 (50.56%)		

*P* < 0.05 was considered statistically significant. IDH1 indicates isocitrate dehydrogenase 1.

According to the results, there may be a correlation between *ASAP3* expression and the molecular subtypes of adult glioma. Meanwhile, *NOTCH3* was found to be expressed low in 102 tumors (48.14%) and high in 109 tumors (51.66%). High expression of *NOTCH3* was predominant in GBM (73.97%) (Table 2). Statistical analysis showed that *NOTCH3* expression was associated with gender, local recurrence, tumor resection, postoperative radiotherapy, WHO classification, and *ATRX* (*P <* 0.05). But there was no relationship with age, ethnicity, tumor size, tumor location, *P53* expression, and *Ki-67* expression in glioma (*P >* 0.05). There was no statistically significant relationship between *NOTCH3* expression and 1p19q codeletion in LGG (*P* = 0.276). In addition, there was no significant difference in the expression of *NOTCH3* between IDH1mut and IDH1wt (*P* = 0.328) (in Table 4). The above results suggested that the expression of *NOTCH3* is not closely related to the molecular subtype of glioma. The correlation between the *ASAP3* and *NOTCH3* protein markers was clarified by Spearman correlation analysis. The *ASAP3* expression was found to be associated with the *NOTCH3* expression (*r* = 0.337; *P* = 0.000) (in Table 5). In adult glioma, the high expression of *NOTCH3* is related to the overexpression of *ASAP3*. Therefore, *ASAP3* and *NOTCH3* are interconnected with each other and associated with the development of adult glioma.

**Table 5:** Relationship between ASAP3 and NOTCH3 expression

Protein	Expression	ASAP3 [*n* (%)]		*r*	*P*-value
		Low	High		
NOTCH3 [*n* (%)]	Low	54 (61.36%)	34 (27.64%)	0.337	0.000
	High	34 (38.64%)	89 (72.36%)		

*P* values were calculated by Pearson’s correlation test. *P* < 0.05 indicates statistical significance.

### 3.7 Prognostic factors for OS and PFS

**Figure 8:**
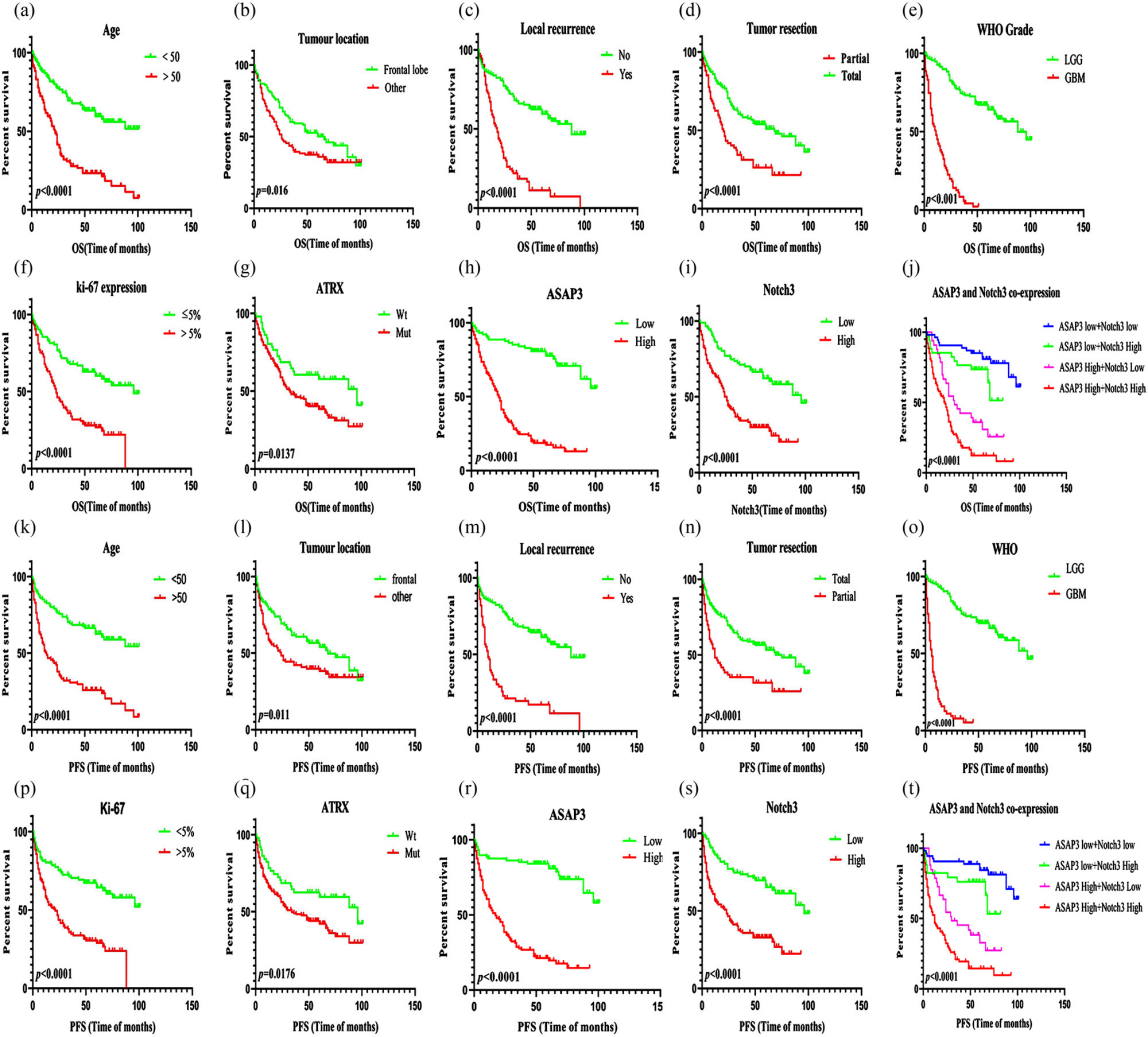
Kaplan–Meier survival analysis for OS and PFS in adult glioma patients. (a–j) The OS of patients in adult glioma with age (a), tumor location (b), local recurrence (c), tumor resection (d), WHO grade (e), Ki-67 expression (f), ATRX expression (g), ASAP3 expression (h), NOTCH3 expression (i), ASAP3 and Notch3 co-expression (j); *P* < 0.05. (k–t) The PFS of patients in adult glioma with age (k), tumor location (l), local recurrence (m), tumor resection (n), WHO grade (o), Ki-67 expression (p), ATRX expression (q), ASAP3 expression (r), NOTCH3 expression (s), ASAP3 and Notch3 co-expression (t); *P* < 0.05.

We constructed univariate and multivariate analyses of OS and PFS in adult glioma patients so that we could evaluate the clinical characteristics of these patients and the prognostic significance of the expression of the *ASAP3* and *NOTCH3* proteins. Two were lost to follow-up in the 211 cases. The Kaplan–Meier method was used to perform a univariate survival analysis. The following prognostic factors influence the OS in adult glioma (Figure 8a–j): Age, tumor location, local recurrence, tumor resection, WHO grade, *Ki-67* expression, *ATRX* expression, *ASAP3* expression, and *NOTCH3* expression. Multivariate analysis (Cox’s proportional hazards regression model) showed that the possible independent prognostic factors for OS were as follows: Age, tumor location, local recurrence, tumor resection, WHO grade, *Ki-67* expression, and *ATRX* expression, *ASAP3* expression, and *NOTCH3* expression (in Table 6).

**Table 6:** Univariable and multivariable analysis for OS and PFS

Clinicopathologic characteristics	OS			PFS		
	Univariate	Multivariate		Univariate	Multivariate	
	*P*-value	*P*-value	HR (95 % CI)	*P*-value	*P*-value	HR (95% CI)
**Age**	<0.0001	<0.0001	3.037 (2.100–4.390)	<0.0001	<0.0001	3.132 (2.135–4.596)
≤50 vs >50						
**Gender**	0.276	0.192		0.142		
Male vs female						
**Ethnic**	0.556	0.889		0.206		
Han vs other						
**Size of the main lesion**	0.899	0.737		0.941		
<3 cm vs ≥3 cm						
**Tumor location**	0.016	0.007	1.481 (1.039–2.110)	0.011	0.0046	1.575 (1.084–2.290)
Frontal vs other						
**Local recurrence**	<0.0001	<0.0001	3.542 (2.342–5.359)	0.000	<0.0001	3.68 (2.371–5.712)
No vs Yes						
**Tumor resection**	0.000	<0.0001	2.221 (1.486–3.318)	0.000	<0.0001	2.307 (1.514– 3.516)
Total vs partial						
**Postoperative radio-chemotherapy**	0.588			0.655		
No vs Yes						
**WHO grade**	0.000	<0.0001	6.28 (3.920–10.06)	0.000	<0.0001	7.046 (4.256–11.66)
LGG vs GBM						
**Ki-67 expression**	0.000	<0.0001	2.541 (1.789–3.609)	0.000	<0.0001	2.702 (1.881–3.880)
≤5% vs >5%						
**P53 expression**	0.328			0.466		
≤5% vs >5%						
**ATRX expression**	0.011	0.014	1.739 (1.177–2.567)	0.014	0.0176	1.725 (1.154–2.579)
Wt vs Mut						
IDH1 expression	<0.0001	<0.0001	5.916 (4.002–8.745)	<0.0001	<0.0001	6.438 (4.278–9.688)
Wt vs Mut						
**ASAP3 expression**	0.000	<0.0001	2.065 (1.418–3.007)	0.000	<0.0001	2.044 (1.383–3.021)
Low vs high						
**NOTCH3 expression**	0.000	<0.0001	2.377 (1.666–3.392)	0.000	0.002	1.857 (1.299–2.657)
Low vs high						
						
**ASAP3 and NOTCH3 co-expression**	0.000	<0.0001		<0.0001		
**(ASAP3 low + NOTCH3 low vsASAP3 low + Notch3 High vs ASAP3 High + NOTCH3 Low vs ASAP3 High + NOTCH3 High)**						

*P* < 0.05 was considered statistically significant.

**Figure 9:**
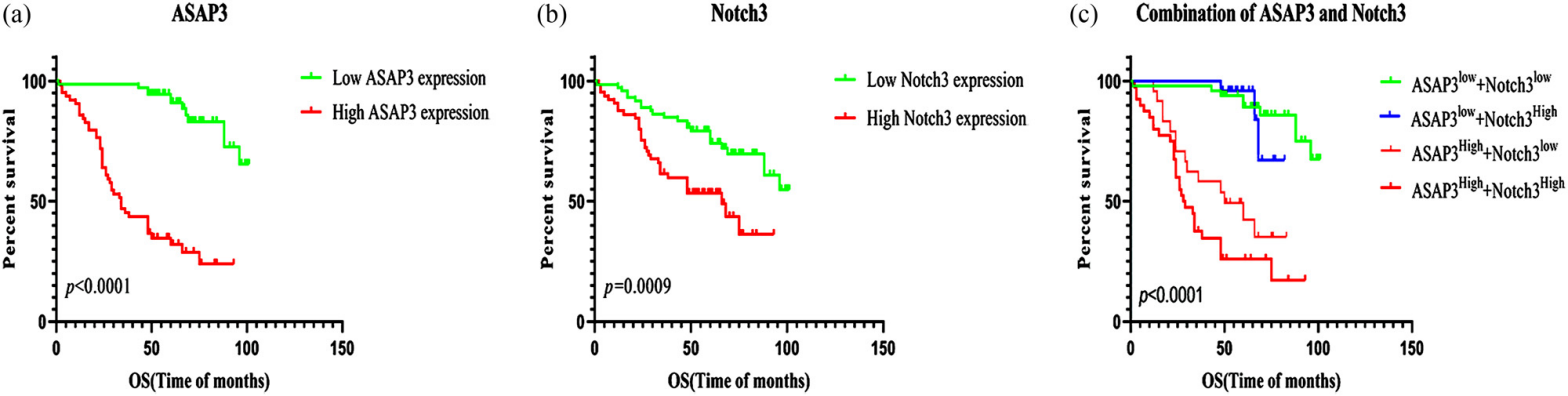
The expression of ASAP3 and NOTCH3 in LGG and their relationship with prognosis. (a) Patients with high ASAP3 expression have a significantly shorter survival time (*P* < 0.0001). (b) Patients with high NOTCH3 expression have a significantly shorter survival time (*P* = 0.0009). (c) Patients with high co-expressions of ASAP3 and NOTCH3 have a shorter OS time (*P* < 0.0001).

In univariate survival analysis, Age, tumor location, local recurrence, tumor resection, WHO grade*, Ki-67* expression, *ATRX* expression, *ASAP3* expression, and *NOTCH3* expression were the significant prognostic factors for PFS in adult gliomas (Figure 8k–t). In multivariate analysis, the prognostic factors were the same as those of OS and *ASAP3* expression. Also, *NOTCH3* expression was independent of prognostic factors for PFS in adult glioma (in Table 6). In conclusion, we analyzed that the independent factors which can predict short OS and PFS in the adult glioma cohort were >50 years old, frontal location, recurrence, tumor total resection, WHO grade IV, *Ki-67* expression >5%, IDH1 expression, and *ATRX* expression. The high expression of *ASAP3* and *NOTCH3* could predict the short OS in adult glioma. Therefore, *ASAP3* and *NOTCH3* may be the potential biomarkers of poor prognosis in adult glioma. According to the results of our study, further analysis of *ASAP3* and *NOTCH3* co-expression in survival analysis in adult gliomas (in Table 7). In the LGG group, the results showed that patients with high *ASAP3* and *NOTCH3* co-expression had shorter OS and PFS. For the GBM group, the expression levels of *ASAP3* and *NOTCH3* were not statistically significant with OS and PFS. In conclusion, there was an inverse relationship between the expression of *ASAP3, NOTCH3* as well as *ASAP3* and *NOTCH3* co-expression with poor OS and DFS in the whole cohort. Cox regression analysis showed that *ASAP3* and *NOTCH3* could be independent prognostic factors for OS in the LGG group (Figure 9).

**Table 7:** Kaplan–Meier survival curves of LGG and GBM patients according to the co-expression of ASAP3 and NOTCH3

Protein expression	OS		PFS	
	LGG (*P*-value)	GBM (*P*-value)	LGG (*P*-value)	GBM (*P*-value)
**ASAP3 expression**				
Low expression vs high expression	<0.0001	0.2717	<0.0001	0.4321
**NOTCH 3 expression**				
Low expression vs high expression	0.0009	0.7815	0.0023	0.1001
**ASAP3 and NOTCH3 co-expression**				
ASAP3 low NOTCH3 low vs ASAP3 low NOTCH3 high vs ASAP3 high NOTCH3 low vs ASAP3 high NOTCH3 high	<0.0001	0.7276	<0.0001	0.519

*P* values were calculated by Pearson’s correlation test. *P* < 0.05 indicates statistical significance.

## 4 Discussion

Glioma in adults remains a disease for which there are no specific therapy targets or prognostic biomarkers. Studying the special biology of glioma through molecular profiling might contribute to new diagnoses that improve therapeutic efficacy. High-throughput sequencing and microarray hybridization technologies have resulted in the development of a variety of gene databases, which have been widely utilized in gene expression quantitative analysis, giving a strong platform for cancer research. The TCGA database was used to derive *ASAP3* expression levels in glioma from the TMA data in the library. The function of *ASAP3* in glioma has not been deeply comprehended in recent years, despite a number of studies on the abnormal expression of RNA and proteins in malignancies.

*ASAP3* was initially found as a widely upregulated gene that leads to cell proliferation in hepatocellular carcinoma. Then, it was identified as a member of the ArfGAP family, which may be involved in the functional role of cell migration in the invasion of normal tissues by cancer cells [6,7]. Subsequently, Ha et al. revealed that *ASAP3* may regulate the filamentous actin of NIH 3T3 cells and perform essential functions in cell migration [16]. *ASAP3* is increased in lung and colorectal cancers with metastasis, which promotes the course of malignant disease and indicates poor patient survival [9]. It is possible that *ASAP3* is involved in the regulation of cell migration and, as a result, the invasion of cancer cells. In response to EGF stimulation, CrkII recruited *ASAP3* to the plasma membrane, where it cooperated with Grb2 and guanine nucleotide exchange factors to promote the recycling of integrin β1 [17]. In addition to regulating EGF-stimulated membrane and cytoskeleton remodeling and the formation of actin-containing stress fibers, *ASAP3* plays an essential role in the process of EGF-elicited cell migration and invasion. *ASAP3* is phosphorylated at Tyr34 in the BAR domain by EGFR receptor kinase in response to EGF stimulation, and CCL18 therapy induces acetylation of Lys311 in *ASAP3*. The location of *ASAP3* at the focal adhesion during cell migration is determined by these post-translational modifications [10]. The interaction between *ASAP3* and *EZRIN* was thought to be important for acid secretion in gastric parietal cells because it controls the movement of K-ATPase-containing tubulovesicles to the apical plasma membrane [18]. On the contrary, upregulation of *ASAP3* also showed a negative effect on the cell adhesion, spreading, and migration on fibronectin. It has been revealed that *ASAP3* was unable to bind to invadopodia in breast cancer cells or podosomes in NIH3T3 mouse fibroblasts. In breast cancer cells transfected with plasmids overexpressing active or inactive *ASAP3*, cellular vinculin or paxillin in focal adhesion and the distribution of invadopodia were not affected. Ha et al. demonstrated that overexpression of *ASAP3* or GAP-inactive mutant *ASAP3*, or *ASAP3* knockdown did not affect vinculin or paxillin distribution, suggesting that *ASAP3* does not affect focal adhesions in cancerous tissues [16]. *MST4* is activated by histamine stimulation, which enhances MST4-*ASAP3* interaction. Furthermore, *MST4* causes a conformational change in *ASAP3*, allowing *ASAP3* to associate with PKA-phosphorylated *EZRIN* at the apical membrane of gastric parietal cells [18].

Previous studies have shown that *ASAP3* is associated with glioma biology [19]. However, there is scarcely any research that investigates whether or not there is a correlation between the expression of *ASAP3* and the prognosis of glioma patients. Through the bioinformatics database, we found that *ASAP3* is closely related to the NOTCH signaling pathway. We analyzed the relationship between the expression of *ASAP3* and *NOTCH3* and the clinicopathological parameters of 211 adult gliomas using TMA. When we compared the expression of *ASAP3* in GBM and LGG, we found that the expression of *ASAP3* in GBM was much higher. This result indicated a favorable connection between the *ASAP3* marker and the glioma grade. Our study demonstrated that the high expression of *ASAP3* in gliomas was closely related to age, high WHO grade, recurrence, resection, postoperative radio-chemotherapy, and *Ki-67* expression ≥10%, suggesting that *ASAP3* was strongly associated with cell proliferation and migration. The high expression rate of *ASAP3* in the IDH1 mutant in LGG was greater than that of the IDH1 wild-type patients. According to the findings of a multivariable study of prognosis, patients whose *ASAP3* expression was high had a considerably worse probability of PFS and OS than patients whose *ASAP3* expression was low. Multivariate prognostic analysis showed that age, tumor site, WHO grade, recurrence, *Ki-67* expression, IDH1 mutant, *ATRX, ASAP3*, and *NOTCH3* expression were shown to be independent predictive determinants of overall survival in gliomas. The high expression of *ASAP3* was positively correlated with the *Ki-67* expression rate ≥10% and the high WHO grade. *Ki-67* is a marker of cell proliferation. IDH1 mutation is associated with a good prognosis and can be used as a predictor of survival. *ASAP3* expression was found to be different in the IDH1 mutant and wild-type. *ASAP3* expression is negatively correlated with IDH1 mutation. Therefore, we speculated that *ASAP3* is related to the proliferation and invasion of adult gliomas, and the effect of *ASAP3* expression on the prognosis of gliomas may be realized by promoting the proliferation of glioma cells and thus changing the tissue grading to achieve the malignant characteristics of tumors. So, there was no connection between *ASAP3* expression and poor OS and PFS in the GBM cohort. Finally, in multivariate analyses between OS and PFS, we determined that *ASAP3* expression was an independent predictive factor for OS. Our findings showed that *ASAP3* may play a significant role in the aggressiveness and progression of gliomas. Accordingly, *ASAP3* may serve as a prognostic indicator and a potential treatment target in adult glioma.

Based on database comparison and literature assessment, the role of the relationship between *ASAP3* and *NOTCH3* in glioma has attracted the research group's interest among the 21 *ASAP3* interacting proteins evaluated in the NOTCH signaling pathway. According to a wide range of studies, NOTCH signaling is involved in regulating biological activity, such as tumor cell adhesion and migration. A wide variety of hematological and solid tumors are associated with the NOTCH signal pathway, which often promotes tumor growth, but it can serve as a tumor suppressor in some cell types [20]. It is still not entirely clear how different NOTCH receptors play a role in the growth of tumors in organisms. The fourmammalian NOTCH paralogs (Notch1–4) are not functionally comparable under all conditions, despite their structural similarities. Studies have shown that binding sites on the promoters of target genes can be linked to different Notch receptors in different ways [20]. The molecular basis for these variances is currently under investigation. Because selectively targeting individual Notch receptors in tumors could reduce treatment side effects, it is important to know which receptor paralogs play key roles in the initial stage and growth of different types of cancer.

Increasing evidence suggests that the NOTCH receptor may be responsible for the development of gliomas. However, the majority of research has focused on the importance of how Notch1-mediated signaling pathways contribute to the development, invasion, and recurrence of glioma tumors. *NOTCH3* was recently verified as a prognostic factor in the regulation of biological activity, including tumor cell adhesion, migration, invasion, and survival [21]. *NOTCH3* belongs to a family of proteins essential for cellular differentiation in a variety of developing tissues. In tumorigenesis, *NOTCH3* has been shown to induce T cell leukemia through the activation of NF-kB. *NOTCH3* amplified is associated with worse survival compared to tumors with non-amplified locus for gliomas in Chinese patients [22]. Rutten et al. also found that *EGFR* and NOTCH signaling have vital functions throughout normal development, and they regularly interact in cooperative and antagonistic manners depending on the cellular context [15]. EGFR opposes NOTCH signaling in various developmental stages, and the NOTCH system can also compensate for hypomorphic alleles of *EGFR* loss of function mutations in Drosophila. Genetic studies demonstrate that *EGFR* and NOTCH signaling pathways interact complexly. For instance, blocking the epidermal growth factor receptor leads to an increase in the number of lung cancer stem cells that are dependent on NOTCH signaling [23]. *EGFR* and LIN-12/NOTCH have conflicting impacts on cell fate determination in *C. elegans vulva* development. The combination of Notch inhibitors with *EGFR* inhibitors, gefitinib or osimertinib, was found to be effective in EGFR tyrosine kinase inhibitor-resistant lung cancer [24]. *NOTCH1* and *EGFR* have been demonstrated to have antagonistic effects in skin cancer, where suppression of *EGFR* results in enhanced differentiation of squamous cell carcinoma cells and increased resistance to apoptosis. These results provide additional support for the combined targeting of *EGFR* and *NOTCH3* signaling to inhibit tumor development. Using bioinformatics and functional tests, it was revealed that the NOTCH pathway is significantly associated with increased *ASAP3* expression in the TCGA GBM cohort (NES = 1.89; *P* = 0.002).

*NOTCH3* is a NOTCH family member that promotes glioma proliferation and invasion [21]. Our studies showed that high *NOTCH3* expression in adult gliomas was related to several factors, including gender, recurrence, tumor resection by surgery or resection, postoperative radio-chemotherapy, higher WHO grade, *Ki-67* expression ≥10%, and *ATRX* expression. Patients with high *NOTCH3* expression showed substantially shorter PFS and OS than patients with low *NOTCH3* expression, indicating that it may emerge as a crucial driver for the malignant development of glioma in a univariate prognostic analysis. Glioma treatment research is now focused on trying to gain a better understanding of the precise processes that are involved in the *NOTCH3* pathway. The chromatin remodeler protein *ATRX* is frequently mutated in H3F3A-mutant pediatric glioblastoma and IDH-mutant grade 2/3 adult glioma [25]. *ATRX* mutation affects DNA damage repair to render these cells more amenable to therapy, which may contribute to the survival advantage of glioma patients with *ATRX* mutations. *NOTCH3* expression was also detected in the majority of vascular endothelial cells associated with tumors, which may facilitate tumor angiogenesis.

*ASAP3* may be combined with *EGFR* to arouse *NOTCH3* expression to promote adult glioma proliferation and invasion. We demonstrated for the first time that *ASAP3* and *NOTCH3* are substantially associated in human glioma samples. The expression of *ASAP3* was an independent prognostic factor for the OS of glioma, and the expression of *ASAP3* was positively correlated with that of *NOTCH3* and *Ki-67* expression rate. We speculated that *ASAP3*, as an upstream factor of the *NOTCH3* signaling pathway, may promote glioma proliferation by regulating the expression of *NOTCH3,* thus affecting the development and prognosis of glioma. Meanwhile, *ASAP3* and *NOTCH3* co-expression correlated with poorer OS and PFS in glioma patients. These results suggest that *ASAP3* may cooperate with *NOTCH3* in the malignant progression of glioma. *ASAP3* and *NOTCH3* co-expression may be a reliable prognostic biomarker. Inhibiting *ASAP3* and *NOTCH3* co-expression may improve the prognosis of glioma patients. However, due to the single experimental method in this study, the mechanism of *ASAP3* in glioma through regulation of the *NOTCH3* signaling pathway needs to be further verified at the cellular level and in animal models so as to further confirm the mechanism of *ASAP3* in glioma. Future studies on the molecular interaction of *ASAP3* and its potential role in the development of glioma will be helpful for a better understanding of the development of this malignant tumor and the clinical tactics of therapy.

## Appendix

**Table A1:** Clinicopathological characteristics in 211 patients with adult glioma

Clinicopathologic characteristics	Total (*N* = 211)	LGG (*n* = 138)	GBM (*n* = 73)	*P*-value
	*n* (%)			
**Age**				
≤50	116 (116/211, 54.98%)	95 (68.84%)	21 (28.77%)	<0.0001*
>50	95 (95/211, 45.02%)	43 (31.16%)	52 (71.23%)	
**Gender**				
Male	123 (123/211, 58.29%)	59 (42.75%)	44 (60.27%)	0.0154
Female	88 (88/211, 41.71%)	79 (57.25%)	29 (39.73%)	
**Ethnic**				
Han	118 (118/211, 55.92%)	76 (55.07%)	42 (57.53%)	0.7319
Other	93 (93/211, 44.08%)	62 (44.93%)	31 (42.47%)	
**Size of the main lesion**				
<3 cm	37 (37/211, 17.05%)	24 (17.39%)	13 (17.81%)	0.9396
≥3 cm	174 (174/211, 82.46%)	114 (82.61%)	60 (43.48%)	
**Tumor location**				
Frontal	108 (108/211, 51.18%)	84 (60.87%)	24 (32.88%)	0.0001*
Other	103 (103/211, 48.82%)	54 (39.13%)	49 (67.12%)	
**Local recurrence**				
No	138 (138/211, 65.41%)	118 (85.51%)	20 (27.40%)	<0.0001*
Yes	73 (73/211, 34.59%)	20 (14.49%)	53 (72.60%)	
**Tumor resection**				
Total resection	142 (142/211, 67.30%)	114 (82.61%)	28 (38.36%)	<0.0001*
Partial resection	69 (69/211, 32.70%)	24 (17.39%)	45 (61.64%)	
**Postoperative radio-chemotherapy**				
No	115 (115/211, 54.50%)	78 (56.52%)	37 (50.68%)	0.418
Yes	96 (96/211, 45.50%)	60 (43.48%)	36 (49.32%)	
**Ki-67 expression**				
≤5%	105 (105/211, 49.76%)	80 (57.97%)	25 (34.25%)	0.001*
>5%	106 (106/211, 50.24%)	58 (42.03%)	48 (65.75%)	
**P53 expression**				
≤5%	128 (128/211, 60.66%)	92 (66.67%)	36 (49.32%)	0.0141*
>5%	83 (83/211, 39.34%)	46 (33.33%)	37 (50.68%)	
**ATRX expression**				
wt	51 (51/211, 24.17%)	39 (28.26%)	12 (16.44%)	
mut	160 (160/211, 75.83%)	99 (71.74%)	61 (83.56%)	0.0564

Statistical analyses were performed by the Pearson *χ* ^2^ test. **P* < 0.05 was considered statistically significant. GBM indicates glioblastoma; LGG, indicates lower grade glioma.

## References

[1] Hu T, Xi J. Identification of COX5B as a novel biomarker in high-grade glioma patients. Onco Targets Ther. 2017;10:5463–70. 10.2147/OTT.S139243. PMC5695267 29180880

[2] Song X, Zhang N, Han P, Moon BS, Lai RK, Wang K, et al. Circular RNA profile in gliomas revealed by identification tool UROBORUS. Nucleic Acids Res. 2016;44(9):e87. 10.1093/nar/gkw075. PMC4872085 26873924

[3] Louis DN, Perry A, Wesseling P, Brat DJ, Cree IA, Figarella-Branger D, et al. The 2021 WHO classification of tumors of the central nervous system: a summary. Neuro Oncol. 2021;23(8):1231–51. 10.1093/neuonc/noab106. PMC8328013 34185076

[4] Gusyatiner O, Hegi ME. Glioma epigenetics: From subclassification to novel treatment options. Semin Cancer Biol. 2018;51:50–8. 10.1016/j.semcancer. 29170066

[5] Okabe H, Furukawa Y, Kato T, Hasegawa S, Yamaoka Y, Nakamura Y. Isolation of development and differentiation enhancing factor-like 1 (DDEFL1) as a drug target for hepatocellular carcinomas. Int J Oncol. 2004;24(1):43–8. 10.3892/ijo.24.1.43. 14654939

[6] Fang Z, Miao Y, Ding X, Deng H, Liu S, Wang F, et al. Proteomic identification and functional characterization of a novel ARF6 GTPase-activating protein, ACAP4. Mol Cell Proteom. 2006;5(8):1437–49. 10.1074/mcp.M600050-MCP200. 16737952

[7] Fan C, Tian Y, Miao Y, Lin X, Zhang X, Jiang G, et al. ASAP3 expression in non-small cell lung cancer: association with cancer development and patients' clinical outcome. Tumour Biol. 2014;35(2):1489–94. 10.1007/s13277-013-1205-1. 24078447

[8] Song X, Liu W, Yuan X, Jiang J, Wang W, Mullen M, et al. Acetylation of ACAP4 regulates CCL18-elicited breast cancer cell migration and invasion. J Mol Cell Biol. 2018;10(6):559–72. 10.1093/jmcb/mjy058. PMC6692856 30395269

[9] Tian H, Qian J, Ai L, Li Y, Su W, Kong XM, et al. Upregulation of ASAP3 contributes to colorectal carcinogenesis and indicates poor survival outcome. Cancer Sci. 2017;108(8):1544–55. 10.1111/cas.13281. PMC5543456 28502111

[10] Zhao X, Wang D, Liu X, Liu L, Song Z, Zhu T, et al. Phosphorylation of the Bin, Amphiphysin, and RSV161/167 (BAR) domain of ACAP4 regulates membrane tubulation. Proc Natl Acad Sci U S A. 2013;110(27):11023–8. 10.1073/pnas.1217727110. PMC3703971 23776207

[11] Monticone G, Miele L. Notch pathway: a journey from notching phenotypes to cancer immunotherapy. Adv Exp Med Biol. 2021;1287:201–22. 10.1007/978-3-030-55031-8_13. 33034034

[12] Matsuura N, Tanaka K, Yamasaki M, Yamashita K, Saito T, Makino T, et al. NOTCH3 limits the epithelial-mesenchymal transition and predicts a favorable clinical outcome in esophageal cancer. Cancer Med. 2021;10(12):3986–96. 10.1002/cam4.3933. PMC8209574 34042293

[13] Pei Y, Li K, Lou X, Wu Y, Dong X, Wang W, et al. miR-1299/NOTCH3/TUG1 feedback loop contributes to the malignant proliferation of ovarian cancer. Oncol Rep. 2020;44(2):438–48. 10.3892/or.2020.7623. PMC7336509 32468036

[14] Bao L, Wang M, Fan Q. Hsa_circ_NOTCH3 regulates ZNF146 through sponge adsorption of miR-875-5p to promote tumorigenesis of hepatocellular carcinoma. J Gastrointest Oncol. 2021;12(5):2388–402. 10.21037/jgo-21-567. PMC8576225 34790400

[15] Rutten JW, Van Eijsden BJ, Duering M, Jouvent E, Opherk C, Pantoni L, et al. Correction: The effect of NOTCH3 pathogenic variant position on CADASIL disease severity: NOTCH3 EGFr 1-6 pathogenic variants are associated with a more severe phenotype and lower survival compared with EGFr 7-34 pathogenic variant. Genet Med. 2019;21(8):1895. 10.1038/s41436-018-0306-z. PMC7608265 30237574

[16] Ha VL, Bharti S, Inoue H, Vass WC, Campa F, Nie Z, et al. ASAP3 is a focal adhesion-associated Arf GAP that functions in cell migration and invasion. J Biol Chem. 2008;283(22):14915–26. 10.1074/jbc.M709717200. PMC2397480 18400762

[17] Song X, Xu W, Xu G, Kong S, Ding L, Xiao J, et al. ACAP4 interacts with CrkII to promote the recycling of integrin β1. Biochem Biophys Res Commun. 2019;516(1):8–14. 10.1016/j.bbrc.2019.05.173. 31182282

[18] Yuan X, Yao PY, Jiang J, Zhang Y, Su Z, Yao W, et al. MST4 kinase phosphorylates ACAP4 protein to orchestrate apical membrane remodeling during gastric acid secretion. J Biol Chem. 2017;292(39):16174–87. 10.1074/jbc.M117.808212. PMC5625048 28808054

[19] Song Y, Shao L, Xue Y, Ruan X, Liu X, Yang C, et al. Inhibition of the aberrant A1CF-FAM224A-miR-590-3p-ZNF143 positive feedback loop attenuated malignant biological behaviors of glioma cells. J Exp Clin Cancer Res. 2019;38(1):248. 10.1186/s13046-019-1200-5. PMC6558706 31186064

[20] Aster JC, Pear WS, Blacklow SC. The varied roles of notch in cancer. Annu Rev Pathol. 2017;12:245–75. 10.1146/annurev-pathol-052016-100127. PMC5933931 27959635

[21] Ulasov IV, Mijanovic O, Savchuk S, Gonzalez-Buendia E, Sonabend A, Xiao T, et al. TMZ regulates GBM stemness via MMP14-DLL4-Notch3 pathway. Int J Cancer. 2020;146(8):2218–28. 10.1002/ijc.32636. 31443114

[22] Shen Z, Hou X, Chen B, Chen P, Zhang Q. NOTCH3 gene polymorphism is associated with the prognosis of gliomas in Chinese patients. Med (Baltim). 2015;94(9):e482. 10.1097/MD.0000000000000482. PMC4553966 25738469

[23] Arasada RR, Amann JM, Rahman MA, Huppert SS, Carbone DP. EGFR blockade enriches for lung cancer stem-like cells through Notch3-dependent signaling. Cancer Res. 2014;74(19):5572–84. 10.1158/0008-5472.CAN-13-3724. PMC4263272 25125655

[24] Bousquet Mur E, Bernardo S, Papon L, Mancini M, Fabbrizio E, Goussard M, et al. Notch inhibition overcomes resistance to tyrosine kinase inhibitors in EGFR-driven lung adenocarcinoma. J Clin Invest. 2020;130(2):612–24. 10.1172/JCI126896. PMC6994195 31671073

[25] Qin T, Mullan B, Ravindran R, Messinger D, Siada R, Cummings JR, et al. ATRX loss in glioma results in dysregulation of cell-cycle phase transition and ATM inhibitor radio-sensitization. Cell Rep. 2022;38(2):110216. 10.1016/j.celrep.2021.110216. PMC8759735 35021084

